# Left anterior descending coronary artery‐left circumflex coronary artery bifurcation angle and severity of coronary artery disease; is there any correlation? A cross‐sectional study

**DOI:** 10.1002/hsr2.2182

**Published:** 2024-06-12

**Authors:** Pejman Mansouri, Ebrahim Nematipour, Nadia Rajablou, Seyyed Mojtaba Ghorashi, Samad Azari, Negar Omidi

**Affiliations:** ^1^ Tehran Heart Center, Cardiovascular Disease Research Institute Tehran University of Medical Sciences Tehran Iran; ^2^ School of Medicine Tehran University of Medical Sciences Tehran Iran; ^3^ Hospital Management Research Center, Health Management Research Institute Iran University of Medical Sciences Tehran Iran; ^4^ Research Center for Emergency and Disaster Resilience Red Crescent Society of the Islamic Republic of Iran Tehran Iran; ^5^ Cardiovascular Imaging Departement, Tehran Heart Center, School of Medicin, Tehran University of Medical Sciences Tehran heart center Tehran Iran; ^6^ Cardiac Primary Prevention Research Center, Cardiovascular Institute, Tehran University of Medical Science Tehran heart center Tehran Iran

**Keywords:** bifurcation angle, calcium score, coronary artery disease, coronary computed tomography angiography, Gensini score

## Abstract

**Background and Aims:**

The aim of this study is to evaluate the association of coronary computed tomography angiography derived (CCTA) plaque characteristics and the left anterior descending coronary artery (LAD) and left circumflex coronary artery (LCX) bifurcation angle with severity of coronary artery disease (CAD).

**Methods:**

All the stable patients with suspected CAD who underwent CCTA between January to December 2021 were included. Correlation between CCTA‐derived aggregated plaque volume (APV), LAD‐LCX angle, remodeling index (RI), coronary calcium score with Gensini score in conventional angiography were assessed. One hundred and twenty‐two patients who underwent both CCTA and coronary angiography were analyzed.

**Results:**

Our analysis showed that the median (percentile 25% to percentile 75%) of the APV, LAD‐LCx angle, and calcium score were 31% (17%−47%), 58° (39°−89°), and 31 (0−186), respectively. Also, the mean ± SD of the RI was 1.05 ± 0.20. Significant correlation between LAD‐LCx bifurcation angle (0.0001−0.684), APV (0.002−0.281), RI (0.0001−0.438), and calcium score (0.016−0.217) with Gensini score were detected. There was a linear correlation between the mean LAD‐LCx bifurcation angle and the Gensini score. The sensitivity and specificity for the cut‐off value of 47.5° for the LAD‐LCX angle were 86.7% and 82.1%, respectively.

**Conclusion:**

There is a direct correlation between the LAD‐LCx angle and the Gensini score. In addition to plaque characteristics, anatomic‐based CCTA‐derived indices can be used to identify patients at higher risk for CAD.

## 1 INTRODUCTION

Coronary angiography (CAG) is the gold standard modality for the diagnosis of coronary artery disease (CAD). However, in some circumstances, it is rational to perform a noninvasive test such as coronary computed tomographic angiography (CCTA) before performing angiography with respect to pretest probability for CAD, risk of complications of CAG, and the ability of CCTA for assessment of plaque characteristics.^1^, ^2^, ^3^ On the other hand, almost one‐third of CAGs comprise normal findings, which justify the use of noninvasive techniques.^4^

Furthermore, CCTA has been recommended as a valuable alternative test for the evaluation of patients with acute chest pain.^5^, ^6^, ^7^ However, the data remains uncertain regarding the potential use of high‐risk plaque characteristics on CCTA in patients with acute chest pain.^8^, ^9^ High‐risk plaques have been noted to have some common characteristics, including plaque burden, positive arterial remodeling, necrotic cores, thin‐cap fibroatheroma, spotty calcifications, low Hounsfield units (HU) attenuation, napkin‐ring sign, and macrophage infiltration.^10^, ^11^, ^12^, ^13^, ^14^, ^15^ Luminal narrowing has also been associated with acute coronary syndrome.^16^ Interestingly, these features have been reported to be associated with an increased risk of adverse cardiovascular events in patients with stable ischemic heart disease.^17^, ^18^, ^19^

Improvement in the image quality of CCTA has led to the precise assessment of plaque characteristics and diagnosis of CAD in the setting of chest pain so that the incidence of non‐assessable segments has decreased from 29% with 4‐line multidetector CT (MDCT) to 4%−6% with 128‐row MDCTs.^20^

In a study by Osborne‐Grinter, Agatston coronary artery calcium score (CACS) couldn't show a significant difference in plaque burden among moderate (100−399 Agatston unit [AU]), high (400−999 AU), and very high (≥1000 AU) groups.^21^ Sama et al., investigated the non‐calcified plaques which have zero calcium score. This study found a 10% prevalence of non‐calcified plaques among zero calcium score patients with chest pain.^22^

According to the previous studies, bifurcation site of left anterior descending artery (LAD) is one of the most common sites for atherosclerotic plaque formation.^23^, ^24^ Different studies have shown different angle cut‐offs to predict atherosclerosis, such as 80° by Sun et al. and 50° in Moon et al. and Cui et al.^25^, ^26^, ^27^

Several studies have been conducted on CAD anatomical criteria and plaque morphology, such as calcium score and LAD bifurcation angle, which have revealed various findings; however, they have failed to accurately explain the difference of ischemic lesions in CCTA as compared to invasive tests. As mentioned above, predicting the behavior of the atherosclerotic plaque could be achieved by more accurate identification of plaque characteristics and, therefore, increasing the accuracy of CCTA. Hence, in the present cross‐sectional study, we aimed to analyze the association between CCTA‐derived plaque characteristics and LAD‐LCX (left circumferential artery) angle with the severity of CAD in CAG.

## 2 METHODS

### 2.1 Study population

In this cross‐sectional study, we retrospectively selected 122 consecutive patients from January to December 2021 who first underwent CCTA and subsequently CAG within 3 months at Tehran Heart Center. Patients with a history of coronary artery bypass graft, percutaneous coronary intervention, pacemaker or implantable cardioverter defibrillator implantation, cardiac resynchronization therapy, valve repair, or replacement were excluded. This study was approved by the Ethical Committee of Tehran University of Medical Sciences regarding the Helsinki Declaration, and informed consent was obtained from all individual participants included in the study.

### 2.2 Baseline measurements, CT angiography, and plaque characterization

Patients' demographic data and risk factors were retrieved using the documented medical electronic files. The CCTA was conducted by Siemens SOMATOM Definition Flash 128‐Slice Dual‐Source CT scanner. CCTA protocol was tailored to each patient on an individual basis, either through automated or manual means. The establishment of intravascular access follows the facility's guidelines, ensuring adequate flow before injection. Adults typically require eighteen‐gauge catheters. The iodine concentration ranges from 270 to 400 mg iodine/cc, with an injection rate of 5−7 cc/s recommended for the injector. Parameters such as slice thickness of 0.6 and reconstruction kernel of 26 were utilized.^28^ A target heart rate of 60 bpm was set for coronary CTA, with beta‐blockers considered as the primary choice for achieving this rate. CCTA findings underwent independent review by a skilled cardiologist and radiologist. In cases of disagreement, a face‐to‐face meeting was arranged for consensus. If consensus was not reached, a third party evaluated the CCTA blindly and made a final decision independently. Calcium score was estimated through the Agatston score with a standard threshold of 130 HU.^29^ Both axial and curved multiplanar reformation images were used for the evaluation of coronary arteries and sclerotic plaques. This enables the Quantitative measurement of coronary artery stenosis in CCTA images using a 2D parametric intensity model.^30^ Three‐dimensional models used in previous studies have some limitations, such as difficult evaluation due to the small size of coronary vessels.^31^

The coronary plaques were divided into three types calcified, non‐calcified, and mixed types according to the amount of calcium. The severity of the stenosis was categorized as minimal, mild, moderate, and severe stenosis.^32^ Furthermore, aggregative plaque volume (APV), LAD artery and left circumflex (LCX) artery angle, remodeling index (RI), and coronary calcium score were measured. APV was described as the ratio of aggregate plaque volume/vessel volume (proximal to the distal part of the lesion), and RI was calculated as the maximal lesion vessel diameter/proximal vessel diameter.^33^ LAD‐LCX angle was measured after determining the centerline vector along the LAD and LCX arteries in Multiplanar reconstruction images in both systole and diastole.

The Gensini score is a comprehensive scoring system that ranks the severity of each coronary artery stenosis based on its degree of stenosis and the significance of the stenosis site. Gensini scores of 1, 2, 4, 8, 16, and 32 were assigned to decreases of 25%, 50%, 75%, 90%, 99%, and full occlusion, respectively.^34^

### 2.3 Study endpoints

The primary endpoint was to assess the correlation of atherosclerotic plaques characteristics and LAD‐LCX angle in CCTA with severity of CAD. The secondary endpoint was to drive a cut point of LAD‐LCX angle to predict severe CAD.

### 2.4 Statistical analysis

All patient's findings were recorded in the checklist and entered into the 22‐SPSS software. Data are presented as mean ± standard deviation (SD), median [IQR 25%−75%], or number (valid percentages). Statistical analyses were provided in descriptive and analytical sections. *χ* ^2^ test was used to analyze the qualitative findings, and an independent *T*‐test was used to compare the quantitative data. Spearman's Rho (SR) test was used for the evaluation of the correlation of the Gensini score with CCTA findings (APV, LAD‐LCx bifurcation angle, Calcium score, and RI). Receiver‐operating characteristic (ROC) curve and the area under the ROC curves (AUCs) were used to assess the diagnostic performance of CAG and CCTA. All tests were one‐sided. The measurements were performed by an expert cardiologist. Interobserver agreement was not applicable. Intra‐observer agreement was assessed by performing repeated measurements by the same observer, but unfortunately, we did not perform a Bland−Altman plot test to resolve the intra‐observer agreement. Probability values of less than 0.05 were considered significant.

## 3 RESULTS

In our study, we assessed the CCTA‐derived indices of 122 patients who underwent CCTA and, subsequently, CAG. Table 1 demonstrates the demographic characteristics of the patients. APV, RI, and calcium score were assessed as the main characteristics of the plaques and LAD‐LCX bifurcation angle in CCTA, and their correlation with the severity of CAD was evaluated. The mean ± SD age of the population was 58.1 ± 12.1 years old, with BMI equal to 23.3 ± 5.4 kg/m^2^, global left ventricular ejection fraction was 52.4% ± 4.8%. Lesions were mostly in LAD (66.8%), followed by RCA (9.3%) and RCA and PDA and PLB (5.2%). Our analysis showed that the median (percentile 25% to percentile 75%) of the APV, LAD‐LCx angle, and calcium score were 31% (17%−47%), 58° (39°−89°), and 31 (0−186), respectively. Also, the mean ± SD of the RI was 1.05 ± 0.20.

**Table 1:** Demographic characteristics of patients.

	Number (%)
Gender (male)	86 (70.5)
Current smoker	30 (24.6)
DM	38 (31.1)
Family history	28 (25.2)
DLP	74 (60.7)
HTN	64 (52.5)
Opium	10 (8.4)

Abbreviations: DLP, dyslipidemia; DM, diabetes mellitus; HTN, hypertension.

**Figure 1:**
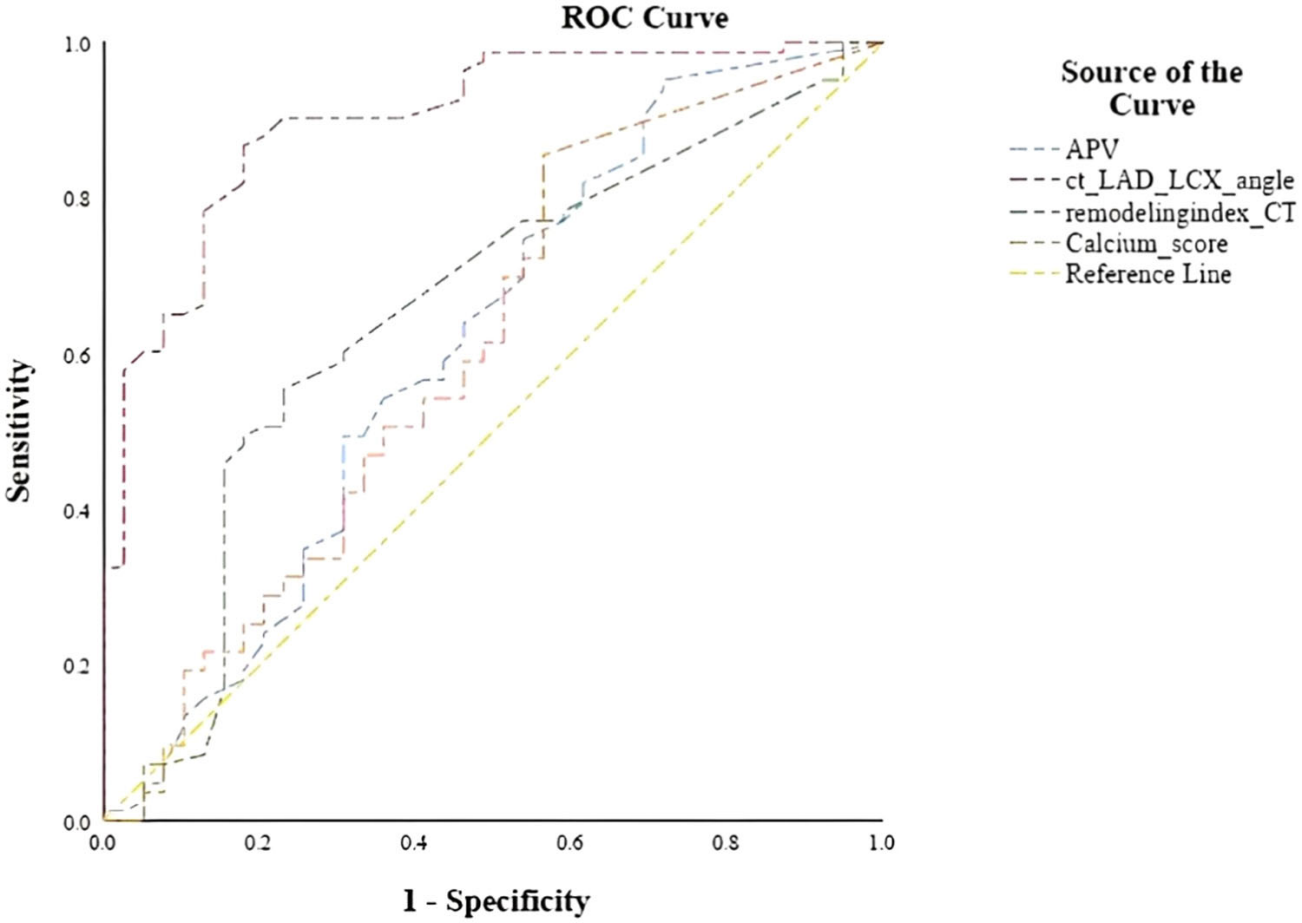
The receiver operating characteristics (ROC) curve for atherosclerotic plaque volume, remodeling index, LAD‐LCX angle, and calcium score. APV, aggregated plaque volume; LAD, left anterior descending; LCX, left circumflex.

SR analysis (*p*‐value, correlation coefficient) showed a significant correlation between LAD‐LCX bifurcation angle (<0.001−0.684), APV (0.002−0.281), RI (<0.001−0.438), and calcium score (0.02−0.217) with Gensini score. After dividing patients into two groups, patients with normal/minimal CAG and patients with CAD, a significant relation between LAD‐LCX angle with the presence of the CAD was seen (*p*‐value: 0.001), but there was no significant relation with calcium score (*p*‐value: 0.55). According to our analysis, as shown in Figure 1, there's a linear correlation between the mean LAD‐LCX bifurcation angle and the Gensini score. The area under the ROC curve for the LAD‐LCX angle was 0.880 (Figure 1) with a cut‐off value of 47.5°, the sensitivity and specificity of 86.7% and 82.1% calculated, respectively. The AUC for APV, RI, and calcium score and related sensitivity and specificity were presented in Table 2.

**Table 2:** The AUC, sensitivity, and specificity for CCTA‐derived features.

	AUC	Standard error	95% of CI	Sensitivity (%)	Specificity (%)
LAD‐LCX angle	0.88	0.037	0.80−0.95	86.7	82.1
APV	0.61	0.059	0.49−0.72	95.2	71.8
RI	0.65	0.055	0.54−0.76	55.4	23.1
Calcium score	0.60	0.058	0.49−0.72	85.5	56.4

Abbreviations: APV, aggregated plaque volume; AUC, area under the curve; CCTA, coronary computed tomography angiography; CI, confidence interval; LAD, left anterior descending; LCX, left circumflex; RI, remodeling index.

## 4 DISCUSSION

Rupture or erosion of the unstable or vulnerable atherosclerotic plaque play a fundamental role in the process of cerebrovascular accidents, acute coronary syndrome, and sudden cardiac death.^35^, ^36^ The CAG lacks the sensitivity and specificity needed to detect the susceptible plaque early enough. When compared to other imaging modalities, such as CCTA or intravascular ultrasound, which may see beyond the lumen and characterize plaque shape or vessel wiggle, the role of CAG is gradually declining.^37^

The present study has two main findings: there was a significant correlation between the LAD‐LCX bifurcation angle and the Gensini score, which means that using the LAD‐LCX angle in CTA has a high diagnostic value for predicting the severity of CAD. The second was to report a cut point of LAD‐LCX angle correlated with more severe CAD.

The findings of this study are in agreement with those of earlier studies on the distribution and morphology of coronary plaques.^38^, ^39^ The LAD, particularly its proximal part, was frequently involved by atherosclerotic plaques, but the LM and the LCx were less frequently impacted. Although more research are needed to assess the potential relevance of these findings, this could be utilized as a guide for analyzing potential impacts of hemodynamics on local features and plaque dispersion.^40^

Earlier research has demonstrated that using the bifurcation LAD‐LCx angle to detect CAD enhances the diagnostic performance, with wide LAD‐LCx angulation related with presence of CAD and high‐risk plaques.^41^, ^42^ When compared to normal coronary arteries, high‐risk plaques were shown to have larger bifurcation angles, showing a link between these angles and the development of high‐risk plaques. Previous studies suggested a significant relation between low wall shear stress (WSS) and endothelial dysfunction atherosclerotic plaque lesions in bifurcations of epicardial coronary arteries.^43^ WSS has long been thought to play an important role in plaque development and progression. Moreover, bifurcation angle alteration is important in the development of atherosclerosis: the larger the bifurcation angle, the more turbulence and hemodynamic impact in the arterial wall.^44^ In a study conducted by Ghafar et al., it was observed that the bifurcation angle was significantly associated with the presence of atherosclerotic plaques at the bifurcation site, with an average angle of 77.32 ± 18.1° compared to 62.24 ± 18.2° in the control group.^30^ Moon et al. suggested that angles greater than 60° between the LAD artery and LCX artery were indicative of a higher presence of atherosclerotic plaques, with a *p*‐value of 0.058.^27^ Juan et al. identified a notable discrepancy in bifurcation angles only among groups with more than 50% stenosis compared to those with unremarkable stenosis.^45^

The precise association between bifurcation angle and CAD, however, has yet to be determined. We found a significant correlation between LAD‐LCx angle measurement and the LAD/LCx involvement in CAG and also a linear correlation between the mean LAD‐LCx bifurcation angle and Gensini score. Our findings of LAD‐LCx angle cut‐off was lower than previous reports, which was between 72 ± 22° and 88.5°.^39^, ^46^, ^47^, ^48^ Cul et al. study^26^ in 2017 on 106 patients suspected of CAD undergoing both CCTA and CAG reported that the bifurcation angle of LAD‐LCx was an independent predictor for significant left coronary stenosis (OR = 1.423, *p* = 0.002). Cui et al. also identified a cut‐off angle of 50°, which is near the cut‐off found in our study. In another study by Sun et al. in 2017,^49^ WSS was noted to increase in the LAD and LCx models with significant stenosis and wider angulation (>80°), but little or no change occurred in most of the coronary models with no significant stenosis and narrower angulation (<80°). As shown in Table 2, the ROC curve for the LAD‐LCX angle yielded an AUC of 0.88, indicating excellent test accuracy according to Mandrekar et al.'s study.^50^ Consequently, further clinical investigations may be warranted to explore the potential significance of a LAD‐LCX angle of 47.5° as a suitable cut‐off point.

Our study contributes additional evidence to support the idea that the angle of bifurcation may serve as a noninvasive means of detecting atherosclerotic plaques, obviating the need for invasive procedures like CAG. Furthermore, our findings suggest that a lower threshold value could potentially enhance sensitivity when applied in larger populations. This study revealed a significant association between calcium score and Gensini score, which is in accordance with recent studies.^51^, ^52^ Gensini score dependency on the percentage of the narrowing of the lumen and coefficient of the affected coronary segment may explain this result. Also, there was a significant correlation between the APV and RI with the Gensini score.

The main limitation of the present study was the cross‐sectional design. Studies with larger sample size and follow‐up of patients would be of value.

## 5 CONCLUSIONS

This study shows the high diagnostic accuracy of using the bifurcation angle in CCTA to predict more severe CAD. However, further research based on a large sample size is needed to support our findings.

## AUTHOR CONTRIBUTIONS

The study design was performed by Pejman Mansouri and Ebrahim Nematipour. Nadia Rajablou collected the data and searched the literature. Statistical analysis was performed by Seyyed Mojtaba Ghorashi. Nadia Rajablou and Samad Azari interpreted the data. Pejman Mansouri prepared the manuscript. Negar Omidi revised the manuscript and the study design. All authors reviewed the manuscript. All authors have read and approved the final version of the manuscript.

## CONFLICT OF INTEREST STATEMENT

The authors declare no conflict of interest.

## ETHICS STATEMENT

The Ethics Committee of Tehran University of Medical Sciences approved the design and conduct of the study. The study was performed in accordance with Helsinki declaration. Informed consent was obtained from all individual participants included in the study.

## TRANSPARENCY STATEMENT

The lead author, Negar Omidi, affirms that this manuscript is an honest, accurate, and transparent account of the study being reported, that no important aspects of the study have been omitted, and that any discrepancies from the study as planned (and if relevant, registered) have been explained.

## Data Availability

The data that support the findings of this study are available from the corresponding author upon reasonable request. The corresponding author had full access to all of the data in this study and takes complete responsibility for the integrity of the data and the accuracy of the data analysis.
